# Decoding the role of chromatin context in the off-target effects of CRISPR gene editing with EGOLD

**DOI:** 10.1038/s41421-026-00889-2

**Published:** 2026-05-13

**Authors:** Hu Feng, Jitan Zheng, Nana Li, Long Xie, Erwei Zuo

**Affiliations:** 1 State Key Laboratory of Genome and Multi-omics Technologies, Shenzhen Branch, Guangdong Laboratory of Lingnan Modern Agriculture, Key Laboratory of Gene Editing Technologies (Hainan), Ministry of Agriculture and Rural Affairs, Agricultural Genomics Institute at Shenzhen, Guangdong, Chinese Academy of Agricultural Sciences, Shenzhen, Guangdong, China; 2 State Key Laboratory for Conservation and Utilization of Subtropical Agro-Bioresources, College of Animal Science and Technology, Guangxi University, Nanning, Guangxi, China; 3 Key Laboratory of Agricultural Animal Genetics, Breeding and Reproduction of Ministry of Education & Key Lab of Swine Genetics and Breeding of Ministry of Agriculture and Rural Affairs, Huazhong Agricultural University, Wuhan, Hubei, China

**Keywords:** Molecular biology, Biological techniques

## Abstract

Despite the power of CRISPR in genome editing, its clinical application is limited by off-target effects; these effects are currently difficult to evaluate at the genome level but are likely to involve chromatin context. Here, we developed the Endogenous Genome-wide Off-target Library Detection (EGOLD) method for high-throughput detection of off-target effects and identification of chromatin context bias in gene editor evaluation. Applying EGOLD to define the off-target characteristics of 17 base-editing tools revealed 2,145,592 total off-targets, with 1236–618,774 events detected per tool. The frequency of off-targets of CRISPR/Cas9 and derivative base editors ranged from 40% to 80% and were strongly influenced by the chromatin context. Using a large-scale endogenous off-target dataset with strict target site conditions to exclude the influence of sequence context, we found that off-target effects occurred in open chromatin genomic regions at a significantly greater frequency than in closed chromatin regions. The incorporation of EGOLD-Seq off-target chromatin context data to train machine learning-based models of gene editor activity substantially improved off-target prediction accuracy. These findings and the accompanying toolkit can guide mechanistic research and the development of safe and precise CRISPR-based tools.

## Introduction

Gene-editing technologies incorporating CRISPR/Cas9 are well established as powerful tools for targeted genetic modification that enable programmable editing with tremendous potential to reverse a variety of genetic disorders^1–4^. However, subsequent evaluations have revealed extensive genome-wide off-target effects mediated by these gene-editing tools^5–10^, and such unintended alterations may result in other genetic disorders, thus negating the benefits of gene therapy^11,12^. Cell type strongly influences the therapeutic efficacy of gene editors^13^, as differences in chromatin context between cells can lead to differences in gene-editing precision and outcomes in otherwise identical genomic sequences. However, how differences in chromatin context influence the rate of off-target effects among gene-editing tools remains unclear.

Previous studies have suggested that the on-target efficiency of CRISPR/Cas9-based systems may be affected by chromatin context^14,15^, as higher editing efficiency is typically observed in more accessible chromatin regions^16^, whereas increased DNA methylation levels are associated with lower efficiency^14^. However, the influence of chromatin context is not easily disentangled from the effects of DNA sequence using evidence obtained from different genomic locations. Similarly, sequence factors have been shown to influence off-target effect rates^17,18^, whereas evidence supporting a relationship between the chromatin context and off-target activity of CRISPR/Cas9 and derivative gene editors is still lacking.

Currently, there are no straightforward, reliable methods for exploring the relationship between chromatin context and off-target effects; thus, new, high-throughput methods for direct off-target information capture from natural genomes are urgently needed to improve safety and facilitate the clinical translation of gene-editing tools. Extensive research has investigated off-target effects in gene editing, with recent efforts focusing on high-throughput capture with synthetic libraries. Lentivirus-integrated libraries often contain diverse characteristics of potential off-target sequences that facilitate detailed off-target analysis for gene-editing tools^14,19^. However, lentiviral off-target libraries, which are randomly integrated into the host cell genome, lack endogenous genome information, such as information concerning chromatin context that might modulate off-target effects in the gene-editing process. Methods for detecting double-strand breaks (DSBs) off-target, such as Digenome-Seq^10^, Guide-Seq^20^, and Circle-Seq^21^^,^ typically yield a limited number of off-target edits. These methods may not provide sufficient data to thoroughly investigate the role of chromatin context or to generate off-target data for base-editing tools.

To understand how chromatin context affects off-target effects, we developed the Endogenous Genome-wide Off-target Library Detection (EGOLD) method for the comprehensive evaluation of off-target base-editing activity, especially specific influences arising from chromatin context. The genome contains a large number of repetitive sequences that commonly exhibit numerous differences at the single-base level. Targeting these loci (on-target sites) can lead to the accumulation of abundant off-target edits at loci containing similar sequences (potential off-target sites) but with distinct chromatin contexts. We constructed EGOLD for rapid analysis of possible correlations between off-target effects and specific chromatin context features. In the current study, we applied EGOLD to evaluate 17 gene-editing tools and consistently observed that the off-target editing frequencies of CRISPR/Cas9 and derivative editors were significantly affected by chromatin context. In particular, compared with activity in closed chromatin regions, the open chromatin state in genomic regions was identified as a key factor associated with increased off-target activity. This study also demonstrates how data acquired through EGOLD can be harnessed to train machine learning prediction models on the off-target effects of different gene-editing tools. This relatively simple, efficient, and accurate method for evaluating genome-wide off-target effects across diverse gene-editing tools can accelerate their development for therapeutic applications.

## Results

### Design of the endogenous genome-wide off-target library detection system

**Fig. 1:**
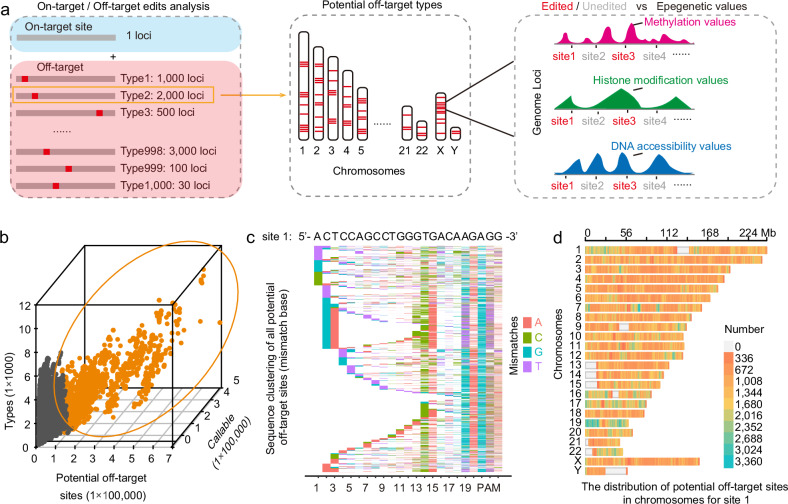
Design of the EGOLD. **a** Workflow for investigating epigenetic mechanisms with EGOLD by comparing chromatin state and epigenetic features between edited and unedited off-target sites with the same DNA sequence. **b** Count, abundance of callable off-targets, and distribution of mismatch types for the 24,116 candidate protospacers. Orange points represent the top 3000 candidate protospacers sorted by callable off-target counts. **c** Heatmap of all potential off-target sites for the site 1 target locus, sorted alphabetically, with mismatches labeled by color. **d** Chromosomal distribution of potential off-target sites for site 1 across the Hg38 genome. “No. of” means “The number of”.

To investigate the role of chromatin context in off-target effects, we need an efficient strategy for detecting endogenous off-targets. To this end, we designed the EGOLD method, which takes advantage of abundant, similar short sequences in the genome. Briefly, we scanned the entire human genome to identify candidate target sequences with extensive similarity to sequences (i.e., 13 nt identical to 23 nt in the target sgRNA) in other genomic regions and different chromatin context features (Fig. 1a). These similar sequences were subsequently assembled into a pool of potential off-target sites for each target locus (Fig. 1a). Each potential off-target site was assigned to a “type” composed of other sites sharing the same sequence. All the genomic sites harboring the same candidate off-target sequence were assigned to the same off-target type. Consequently, sites within the same type are sequence-identical but reside at different genomic locations and are embedded in distinct chromatin contexts. The biological rationale for this classification is to decouple sequence effects from chromatin effects. By grouping sequence-identical off-target sites into a single type, we are able to control for sequence composition while systematically examining how chromatin context influences off-target editing outcomes. For instance, Type 2 included 2000 potential off-target sites that were completely sequence-identical but were scattered throughout the genome and embedded in distinct chromatin contexts across different genomic locations. After editing a given target sequence, we collected editing data for thousands of potential off-target sites with diverse chromatin contexts from the corresponding pool (Fig. 1a and Supplementary Fig. S1a). Analysis of correlations between off-targets and specific chromatin context features was then used to guide functional investigations of the role of chromatin context in off-target editing effects.

Specifically, this initial screen for candidate target genomic loci was performed with eight primary steps (see Materials and methods) to reduce the likelihood of on-target edits disrupting the cell cycle or eliminating whole chromosomes^22^, potentially resulting in death of the host cell during editing (Supplementary Fig. S1a). We applied this relatively stringent screening process to 223,349,907 candidate sites, which yielded 24,116 potentially viable candidate target loci. These candidate target loci all had between 5000 and 629,879 potential off-target sites (Supplementary Fig. S1b), which included a wide range of mismatch types that collectively contained abundant epigenomic information and had highly variable genomic distributions. These potential off-target sites were also detected by whole-genome sequencing (WGS) based on unique features in their specific sequence context (i.e., they were callable in the analysis process; Supplementary Fig. S1c, d). Consequently, we sorted the candidate protospacers based on their number of callable predicted off-target sites and selected the top 3000 positions as preliminary candidate protospacers (Fig. 1b and Supplementary Table S1). As with the larger set of candidates, the potential off-targets for this subset also included a diverse range of mismatch types with broad and varying genomic distributions across different chromosomes (Supplementary Fig. S1e, f). By targeting a single endogenous locus from this filtered subset, we were able to gather off-target editing information from an extensive pool of possible sites, enabling a comprehensive analysis of the off-target characteristics of a variety of gene-editing tools.

To test EGOLD as a method for collecting off-target editing data for different Cas9 variants, we first randomly selected 10 target sites from the pool of 3000 candidates (Supplementary Fig. S1g and Tables S2 and S3). We then assessed the on-target efficiency of a panel of Cas9-based editors that included wild-type Cas9^23^ (Cas9) alongside six high-fidelity variants: eCas9^24^, HiFiCas9^25^, HypaCas9^26^, SuperFiCas9^27^, OptiCas9^28^, and Sniper2LCas9^29^. Additionally, we included one noteworthy Cas9 variant, RYCas9^30^, which has nearly protospacer adjacent motif (PAM)-relaxed editing features. In our preliminary test results, all of the Cas9 variants exhibited editing activity at site 1 (5’-ACTCCAGCCTGGGTGACAAG + AGG-3’ sgRNA+PAM; Supplementary Fig. S2a). We closely examined the sequencing data for this editor with site 1 and found a wide variety of mismatch types that were broadly distributed across multiple chromosomes (Fig. 1c, d). This target site was thus chosen for the next EGOLD step. To avoid possible biases associated with single-site data, we also included sites 2, 5, 6, 7, and 10 in off-target detection analyses for some editing tools (Supplementary Fig. S2b).

### EGOLD analysis of sgRNA-dependent off-target edits

**Fig. 2:**
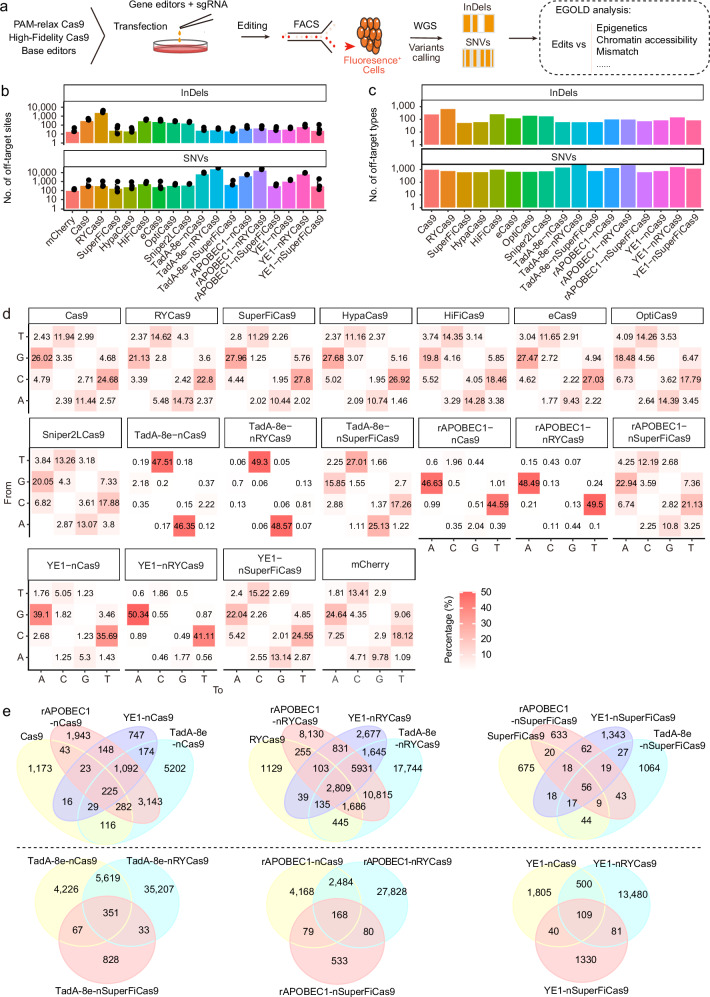
Validation of EGOLD with Cas9-based editors. **a** Experimental workflow for detecting sgRNA targeting site 1-dependent off-targets of various editing tools. **b** Number of sgRNA-dependent InDels or SNVs off-targets for 17 editing tools targeting site 1, with mCherry as the control. **c** Number of InDels or SNVs off-target types for 17 editing tools targeting the site 1 locus. **d** Heatmap illustrating the pattern of single-nucleotide mutations associated with the 15 editing tools. “From” indicates the base on the reference genome, while “To” indicates the base after mutation. **e** Venn diagram of overlapping off-target sites of Cas9 variants and base-editing tools. “No. of” means “The number of”.

We next investigated the off-target effects of the Cas9 variants through the EGOLD pipeline. Cas9 variants, including Cas9, eCas9, HiFiCas9, HypaCas9, SuperFiCas9, OptiCas9, Sniper2LCas9, and RYCas9, were used to target site 1 to obtain off-target data, with mCherry as the control. Briefly, we co-transfected plasmids expressing individual Cas9 variants and sgRNA targeting site 1 (Fig. 2a) and then isolated positively transfected cells by fluorescence-activated cell sorting (FACS) at 48 h post-transfection, extracted their DNA for WGS, and performed off-target analysis (Fig. 2a). The results revealed that the numbers of insertions and deletions (InDels) among off-target edits ranged from 11 to 2799 across the different Cas9 editors (Fig. 2b), and these changes were accompanied by a rich variety of sequence features at these off-target sites (Fig. 2c).

Among the Cas9 variants, RYCas9 mediated the greatest number of off-target effects, whereas SuperFiCas9 mediated the fewest off-target effects and presented > 25% on-target efficiency, indicating a low off-target rate and high fidelity in editing tools. We subsequently selected these two Cas9 variants, along with Cas9, for fusion with the rAPOBEC1^31^ or YE1^6^ cytosine editors or the TadA-8e^32^ adenine editor. EGOLD analysis revealed numerous off-target single-nucleotide variants (SNVs) edits (478 ± 332–23,854 ± 2001, mean ± S.D.) among the different treatment groups, including a diverse range of mismatch types (82–2368) (Fig. 2b, c). The mutation distribution pattern revealed that the off-target effects induced by Cas9 were not biased, whereas the base-editor groups presented clear C- > T/G- > A (CBEs) or A- > G/T- > C (ABEs) substitution patterns, indicating that the off-target data from EGOLD are reliable (Fig. 2d and Supplementary Fig. S2c). In addition, we found that the off-target site counts and off-target edits were highly consistent across replicates, enabling clear distinctions in off-target effects between editors (Supplementary Fig. S3a).

Notably, we observed relatively low overlap in off-target edits between Cas9 variants and their respective derivative base editors incorporating TadA-8e, rAPOBEC1, or YE1 (Fig. 2e). Compared with Cas9, TadA-8e-, rAPOBEC1-, and YE1-nCas9 contained 93.65%, 91.67%, and 88.06% unique off-target sites, respectively. Compared with RYCas9, TadA-8e-, rAPOBEC1-, and YE1-nRYCas9 contained 87.69%, 84.12%, and 78.22% unique off-target sites, respectively. In parallel, for SuperFiCas9, TadA-8e-, rAPOBEC1-, and YE1-nSuperFiCas9 had 90.15%, 88.02%, and 93.01% unique off-target sites, respectively. Likewise, the same deaminase fused to different Cas9 nickase variants also showed low overlap in off-target edits. The TadA-8e fusion with -nCas9, -nRYCas9, and -nSuperFiCas9 contained 41.18%, 85.43%, and 64.74% unique off-target sites, respectively. The rAPOBEC1 fusions with -nCas9, -nRYCas9, and -nSuperFiCas9 contained 60.41%, 90.06%, and 61.98% unique off-target sites, respectively. The YE1 fusions with -nCas9, -nRYCas9, and -nSuperFiCas9 contained 73.55%, 95.13%, and 85.26% unique off-target sites, respectively (Fig. 2e). These results indicated that sgRNA-dependent off-target edits may vary even within the same Cas9 variant architecture or among different base editors that employ the same deaminase. We also observed relatively low overlap in off-target edits between the HEK293T and K562 cell lines (Supplementary Fig. S3b). Overall, these results highlight the ability of EGOLD to extract information from editing data, especially regarding the nature and extent of off-target effects.

In light of the off-target editing data obtained with EGOLD above, we next sought to compare the off-target characteristics of different Cas9 variants in parallel. In the tested group, the high editing efficiency at target sites but low off-target effects suggested that SuperFiCas9 variants could retain efficiency comparable to that of Cas9 variants while reducing the risk of mutation in other genes (Supplementary Fig. S4a, b). To characterize the distinct properties of off-target activity caused by different Cas9 variants, we next explored the specific features of the PAM context, mismatch counts, and mismatch position. To investigate possible PAM context bias of Cas9 variants, we collected off-target counts, editing efficiency at off-target loci, and the proportions of off-target edits corresponding to “NGG” and “non-NGG” PAM types (Supplementary Fig. S4c). Unexpectedly, we found that “non-NGG” off-target edits were abundant across the different editor groups (45–5539) and were especially prevalent in cells treated with RYCas9, whose number and percentage of edits were greater at “non-NGG” loci (0.20%, 5539/2,783,125) than at NGG loci (0.02%, 125/684,540). In contrast, we observed a bias toward the “NGG” PAM context in the other Cas9 variants (Supplementary Fig. S4c, d). We subsequently analyzed the frequency of mismatches at off-target sites in each group and found that tolerance could reach up to four mismatches at most off-target sites for most Cas9 variants. However, the editing activity significantly decreased at off-target sites containing four or more mismatches (Supplementary Fig. S4e). In contrast, RYCas9 exhibited markedly greater tolerance for mismatches at off-target sites (Supplementary Fig. S4f). Analysis of mismatch positions within off-target sequences revealed a greater likelihood of mismatches occurring on both sides of the sgRNA target sequence in Cas9 variants (Supplementary Fig. S4f). With respect to the off-target edits of base editors, our findings revealed that the off-target characteristics, including PAM context, mismatch count tolerance, and mismatch position, were similar among the variants (Supplementary Fig. S5).

### An open chromatin state promotes the off-target activity of CRISPR/Cas9-derived nucleases

On the basis of these large-scale off-target data from EGOLD, we next analyzed the role of chromatin context in the off-target base changes mediated by Cas9 variants. For this purpose, we focused on thirteen chromatin features of the off-target sites to investigate whether and which of these features might promote, hinder, or not affect off-target activity. These features included histone modifications (i.e., H3K9me3, H3K4me1, H3K36me3, and H3K27ac), DNA methylation, chromatin accessibility (evaluated by ATAC-seq, DNase-seq, and ChIP-seq data from RNAP-II, TCF27, ELK4, TCF7L2, and TRIM28), and RNA-seq data (FPKM, with higher values suggesting greater chromatin accessibility). These data were primarily sourced from the ENCODE database (Supplementary Table S4).

**Fig. 3:**
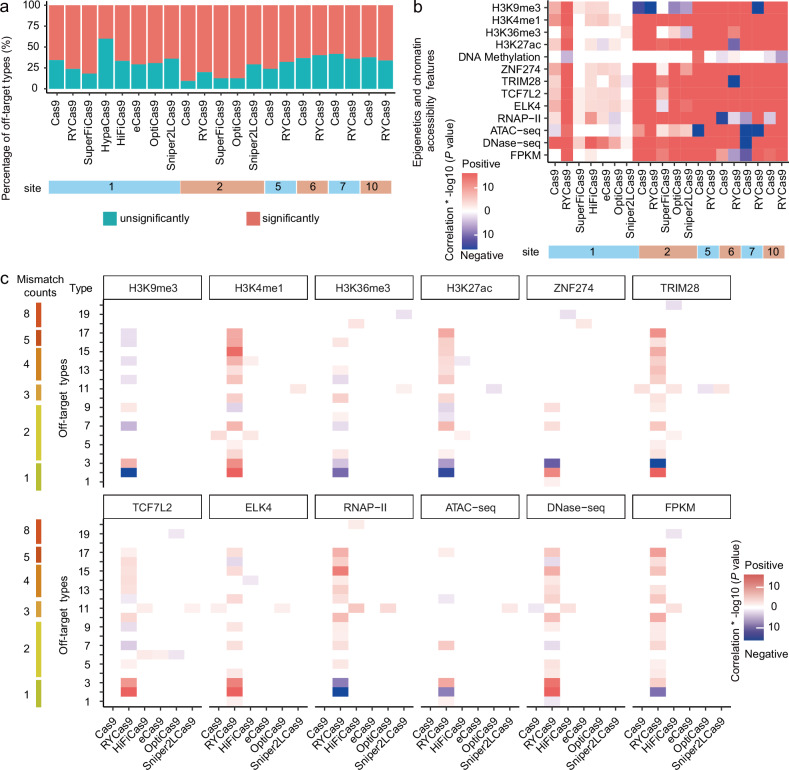
Effects of epigenetic features and chromatin accessibility on the off-target activity of Cas9 variants. **a** Percentage of off-target types with significant and non-significant associations. Off-target types were considered “influenced” by chromatin context if they were significantly associated with any specific epigenetic feature or chromatin accessibility (*P* < 0.05, Wilcoxon test); the minimum off-target number for each type was ≥ 10. **b** Heatmap of *P* values for different epigenetic or chromatin accessibility features in correlation analyses with edited and unedited off-target sites for each gene editor (*P* < 0.05, Wilcoxon test). Red reflects positive correlations; blue reflects negative associations. **c** Relationships between off-target effects and different epigenetic features or degrees of chromatin accessibility for each Cas9-variant gene editor (*P* < 0.05, Wilcoxon test). Red reflects positive correlations; blue reflects negative associations.

Briefly, we screened off-target types (i.e., those with the same sequence but different genomic locations) with ≥ 10 edits and performed Wilcoxon tests between each chromatin feature at edited and unedited sites within that type for sgRNA site 1. Surprisingly, we found that a large percentage of the off-target types (65.63%, 21/32) for Cas9 were significantly (*P* < 0.05) correlated with at least one chromatin feature (Fig. 3a). Similarly, a large proportion of off-target types for RYCas9 (76.25%, 61/80), SuperFiCas9 (81.82%, 9/11), HypaCas9 (40.00%, 6/15), HiFiCas9 (66.67%, 26/39), eCas9 (70.83%, 17/24), OptiCas9 (69.23%, 18/26), and Spinper2LCas9 (64.00%, 16/25) were also significantly (*P* < 0.05) correlated with at least one chromatin feature (Fig. 3b). Analysis of sgRNA site 2 also revealed that a large proportion of off-target types for Cas9 (90.70%, 39/43), RYCas9 (80.17%, 97/121), SuperFiCas9 (87.50%, 7/8), and Spinper2LCas9 (70.83%, 17/24) were significantly (*P* < 0.05) correlated with at least one chromatin feature (Fig. 3b); this pattern of significant correlation with at least one chromatin feature was consistently observed for Cas9 and RYCas9 off-targets associated with sgRNA sites 5, 6, 7, and 10 (*P* < 0.05) (Fig. 3b).

To evaluate overall differences in chromatin context that could lead to off-target base changes, we compared chromatin context features between edited and unedited off-target sites for each significant type mentioned above. Among the chromatin context features, DNase-seq values (which reflect chromatin accessibility) strongly correlated with off-target edits in both the Cas9 group and the other base-editor groups (*P* < 0.05, 100%, 20/20), whereas DNA methylation had a relatively minor effect (*P* < 0.05, 30%, 6/20) (Fig. 3b). Among the Cas9 derivatives, the majority of the analyzed chromatin features significantly contributed to off-target editing by RYCas9 (*P* < 0.05, 100%, 13/13), whereas relatively few chromatin features influenced Sniper2LCas9 off-target edits (*P* < 0.05, 30.77%, 4/13) (Fig. 3b).

To further investigate the relationship between off-target edits and chromatin features in Cas9 derivative gene editors, we assembled a set of 20 off-target types, each with at least 50 edited off-target sites, to minimize sampling bias, that significantly differed in at least one chromatin feature. This analysis revealed that in terms of the number of affected sites, H3K9me3, in particular, was negatively correlated with most off-target types, whereas the other features were positively correlated with most off-target types (Fig. 3c and Supplementary Fig. S6). Interestingly, among off-target types with one nt mismatch from the target sgRNA, the strength of positive correlations varied between off-target edits and chromatin features, such as histone modifications (H3K9me3, H3K36me3, and H3K27ac), or chromatin accessibility (ATAC-seq, DNase-seq, RNAP-II, TCF27, ELK4, TCF7L2, TRIM28, and ZNF274). These results suggest that chromatin context might have a stronger positive influence on off-target activity at potential sites with more than three mismatched nucleotides. Similar overall trends were observed in comparisons of off-target types at sites 2, 5, 6, 7, and 10 (Supplementary Fig. S6 and Tables S5–S8). In addition to providing proof-of-concept evidence that EGOLD could be used for side-by-side comparisons of off-target characteristics among Cas9 variants, these findings indicated that the chromatin context can significantly promote off-target editing activity.

### An open chromatin state promotes the off-target activity of base editors

**Fig. 4:**
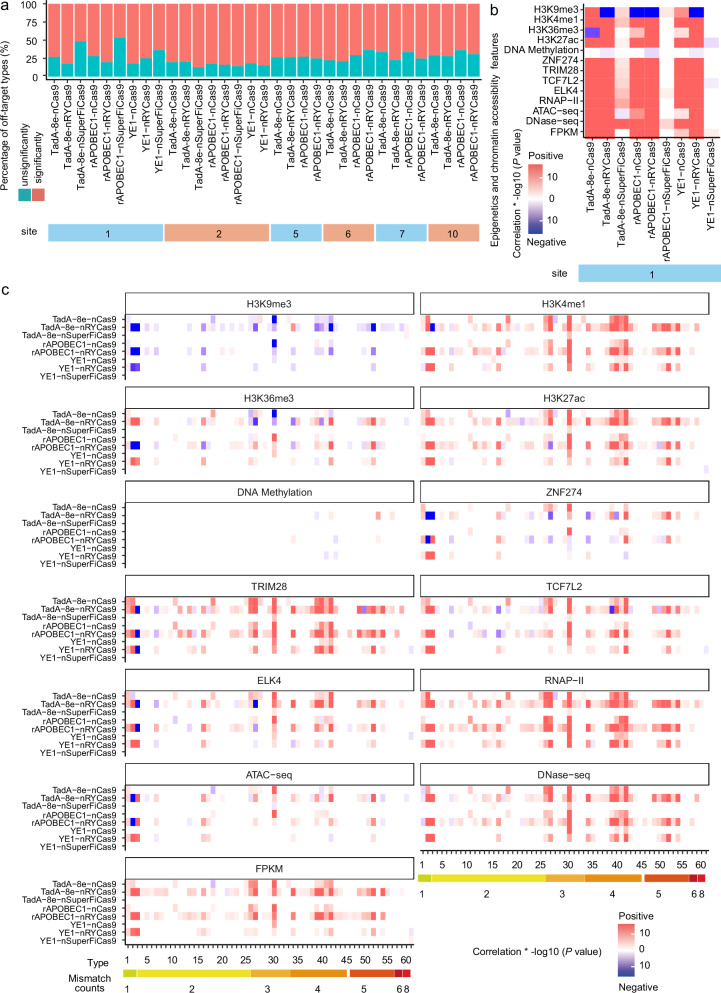
Effects of epigenetic features and chromatin accessibility on the off-target activity of base-editing tools. **a** Percentage of off-target types with significant and non-significant associations. Off-target types were considered “influenced” by chromatin context if they were significantly associated with any specific epigenetic feature or chromatin accessibility (*P* < 0.05, Wilcoxon test); the minimum off-target number for each type was ≥ 10. **b** Heatmap of *P* values for different epigenetic or chromatin accessibility features in correlation analyses with edited and unedited off-target sites for each gene editor (*P* < 0.05, Wilcoxon test). Red reflects positive correlations; blue reflects negative associations. **c** Relationships between off-target effects and different epigenetic features or degrees of chromatin accessibility for each gene editor (*P* < 0.05, Wilcoxon test). Red reflects positive correlations; blue reflects negative associations.

We then applied the EGOLD off-target dataset to examine whether and which of the thirteen chromatin features might also influence off-target base-editor activity associated with different deaminases using the same workflow as that used for different CRISPR/Cas9 nucleases. Briefly, we collected off-target types with ≥ 10 edits and performed Wilcoxon tests between each chromatin feature and edited or unedited off-targets for each type associated with sgRNA site 1. Among the tested base editors, the proportion of off-targets varied from 46.67% to 82.35% of the complete potential off-target pool. In particular, TadA-8e-, rAPOBEC1-, and YE1-nRYCas9 fusions presented the highest rates of off-target activity, with 82.35% (182/221), 80.29% (167/208), and 75.00% (90/120) of types significantly (*P* < 0.05) affected by at least one chromatin feature, respectively. In contrast, TadA-8e-, rAPOBEC1-, and YE1-nSuperFiCas9 fusions had the lowest off-target activity and the fewest off-target types significantly associated with at least one chromatin feature, accounting for 52.00% (13/25), 46.67% (7/15), and 63.64% (14/22), respectively. In line with the results of the results of the CRISPR/Cas9 off-target analyses, TadA-8e-, rAPOBEC1-, and YE1-nCas9 fusions presented intermediate off-target levels but relatively high proportions of off-target types significantly correlated with at least one chromatin feature, including 72.97% (54/74), 71.83% (51/71), and 81.82% (27/33) of the types, respectively (Fig. 4a). Further analysis revealed that TadA-8e-, rAPOBEC1- and YE1-nCas9/-nRYCas9 fusions had intermediate off-target levels associated with sgRNA sites 2, 5, 6, 7, and 10, and a relatively high proportion of the detected off-target types were significantly correlated with at least one chromatin feature (Fig. 4a). These results suggest that chromatin context significantly affects the off-target activity of base editors.

To evaluate overall differences in chromatin context among edited and unedited off-target sites, we compared chromatin context profiles between edited and unedited sites within each off-target type. Among base editors, TadA-8e-, rAPOBEC1-, and YE1-nRYCas9 fusions were significantly influenced by all 13 chromatin features (*P* < 0.05, 100.00%, 13/13), whereas YE1-nSuperFiCas9 fusions were least affected by chromatin features (*P* < 0.05, 23.08%, 3/13) (Fig. 4b). Among the different chromatin features, DNA methylation and H3K9me3 were primarily negatively correlated with off-target activity, whereas other chromatin context features were positively correlated with off-target edits. The same comparative analysis with edited data from sgRNA sites 2, 5, 6, 7, and 10 was similar but with a higher proportion of significant differences (Supplementary Fig. S7).

To further investigate the relationship between chromatin features and the off-target activity of base editors with different deaminases, we compiled a set of 61 off-target types again, each with at least 50 edited off-target sites, to reduce sampling bias, which significantly differed in at least one chromatin feature (Fig. 4c). As base editors generally had higher off-target numbers than their corresponding Cas9 nuclease alone did, more types were available for analysis. In line with our above analyses, DNA methylation and H3K9me3 features were associated with unedited sites, supporting a negative regulatory role in off-target activity, whereas other chromatin features were positively correlated with the frequencies for most off-target types. Similar to our observations in Cas9 variants, the influence of chromatin context increased with the number of mismatched base pairs compared with that of the sgRNA. Comparisons of sites 2, 5, 6, 7, and 10 off-target editing data revealed similar overall trends for each chromatin feature (Supplementary Tables S5–S9). This proof-of-concept evidence supported the use of EGOLD for the comprehensive evaluation of off-target activity characteristics and revealed that chromatin context is a major factor in promoting or suppressing off-target editing at different genomic sites.

### EGOLD offers off-target datasets to support machine learning models for off-target prediction

**Fig. 5:**
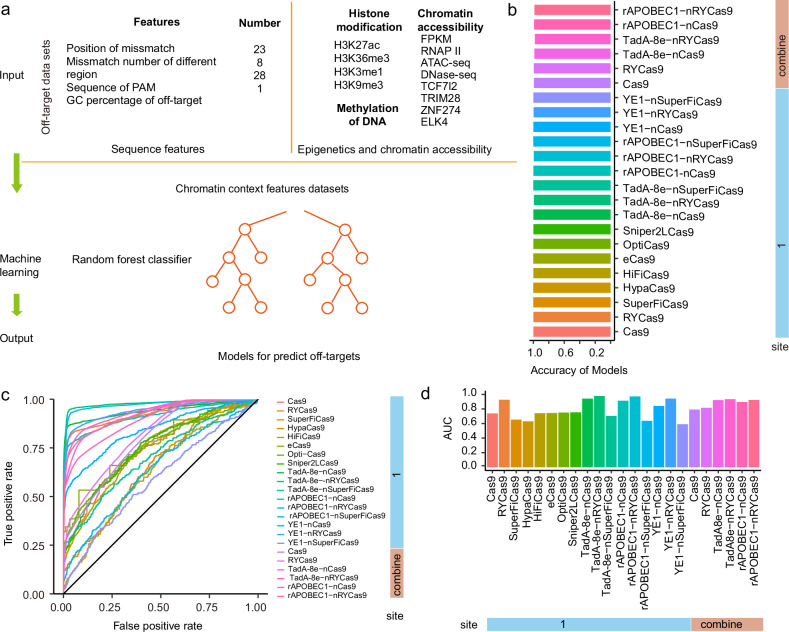
EGOLD provides extensive data for training off-target prediction models. **a** Schematic diagram of machine learning model construction with EGOLD datasets. **b** Predictive accuracy of the models for each gene editor. **c** Receiver operating characteristic curve (ROC) of editing tools. **d** Area under the ROC curve (AUC) of the editing tools.

We next assessed whether edit feature data obtained via the EGOLD method could be used to train tree-based ensemble machine learning models to predict the effects of Cas9 variants and derivative base editors using off-target data (Fig. 5a). For this purpose, we extracted 75 off-target editing characteristics as input features for training (Supplementary Table S10), 62 of which were sequence-derived information, e.g., mismatch details and mutation patterns, in comparison with reference target sequences, and 13 of which were related to epigenetic feature regulation, including histone modifications and DNA methylation, and chromatin accessibility, such as ATAC-seq, DNase-seq, RNAP-II, TCF27, ELK4, and TCF7L2, and TRIM28. These data were sourced from the ENCODE database (Supplementary Table S4). These feature inputs were given the same weight initialization settings and then subjected to classification and regression using random forest modeling to identify the optimal features within random subsets of input features, which should enable the construction of more robust predictive models.

The EGOLD edit feature datasets were then randomly divided into training (80%) and testing (20%) datasets. Different Cas9 variants and the same set of base editors as above were iteratively tested with different combinations of hyperparameters (e.g., number of estimators, maximum depth, learning rate, subsample, colsample_bytree, etc.), and the highest accuracy model was then saved. Comparisons with observed edit data for site 1 revealed that the models of each Cas9 variant provided relatively high predictive accuracy in the testing datasets: Cas9 (99.92%), RYCas9 (99.82%), SuperFiCas9 (99.97%), eCas9 (99.96%), HypaCas9 (99.96%), HiFiCas9 (99.89%), OptiCas9 (99.94%), and Sniper2LCas9 (99.94%) (Fig. 5b). Similarly, the base-editor activity models also provided high-accuracy predictions in the test datasets for the TadA-8e-, rAPOBEC1-, and YE1-fusions with nCas9 (99.83%, 99.84%, and 99.92%, respectively), nRYCas9 (99.25%, 99.39%, and 99.65%), and nSuperFiCas9 (99.95%, 99.96%, and 99.93%). To improve the generalizability of the models, we combined off-target data from sites 1, 2, 5, 6, 7, and 10 into a unified dataset. The model accuracies on this combined dataset for Cas9, RYCas9, TadA-8e-nCas9, TadA-8e-nRYCas9, rAPOBEC1-nCas9, and rAPOBEC1-nRYCas9 were 99.25%, 99.82%, 98.55%, 97.87%, 99.00%, and 98.40%, respectively. We also found that the area under the receiver operating characteristic (ROC) curve (AUC) scores for each model were proportional to the amount of off-target data, with higher amounts of off-target data resulting in relatively higher AUC values (Fig. 5c, d). These results suggest that EGOLD could be used to efficiently train machine learning models that predict off-target effects with high accuracy.

To assess the contributions of different features to the predictive accuracy of off-target models for Cas9 variants and base-editor combinations, we extracted feature weights from each model (Supplementary Fig. S8). Among the 75 input features, the number of mismatched bases at the target site exerted the strongest influence on off-target effects across all the gene-editing tools, with importance scores ranging from 0.023 to 0.347. Notably, individual epigenetic or chromatin features generally exhibited modest importance when considered in isolation. However, when aggregated, chromatin-related features collectively contributed substantially to the overall predictive performance, with cumulative importance scores ranging from 0.039 to 0.193 (Supplementary Fig. S8).

## Discussion

The state of chromatin, including both heterochromatin and relaxed euchromatin, is regulated by various transcriptional regulatory mechanisms and epigenetic mechanisms, including modifications such as methylation, acetylation, and phosphorylation. Although DNA is more readily accessible to the Cas9 protein in regions of open chromatin, the overall influence of the chromatin state on on-target editing efficiency and off-target activity in CRISPR/Cas9-mediated genome editing remains relatively unexplored. Previous studies of off-target effects have focused primarily on factors within DNA sequences but have paid little attention to the impact of chromatin state, owing in large part to the difficulty of obtaining high-throughput endogenous off-target data. Moreover, isolating the influence of sequence factors through the use of uniform off-target sites has also proven challenging. The EGOLD method overcomes this issue by using native genomic sequences and covering a sufficient diversity of potential off-target sites to enable monitoring of base-editing activity at the same sites under different conditions of epigenetic regulation and chromatin accessibility.

The evidence obtained in our current study suggests that both epigenetic features and chromatin accessibility influence off-target editing activity. By comparing sequencing data from the same off-target sites via EGOLD, we compared epigenetic profiles and chromatin states between edited and unedited sites with the same DNA sequence (Fig. 1a). This capability enables thorough investigation of the relationship between chromatin context and off-target activity. Our findings suggest that off-target effects are more likely to occur in open chromatin genome regions than in closed chromatin regions with the same DNA sequence. Differences in chromatin context between edited and unedited potential off-target sites at sites 1, 2, 5, 6, 7, and 10 in EGOLD show consistent patterns. Interestingly, edits involving perfectly matched or single mismatches with the sgRNA appear to be less affected by epigenetic features. However, as the number of mismatched base pairs increases, off-target edits exhibit an increasingly stronger correlation with epigenetic features. A higher mismatch burden is expected to destabilize sgRNA-Cas9 binding to genomic DNA, under which conditions off-target activity appears to be more closely associated with local chromatin accessibility. Importantly, this observation reflects a correlation rather than a causal relationship and may arise from the combined effects of chromatin state, DNA repair processes, and other regional genomic features that modulate editing outcomes when sgRNA–DNA binding becomes less stable.

Lentivirus-integrated libraries^33,34^ typically include extensive mismatch and mutation types for a given target sequence and are therefore commonly used for comprehensive characterization of the sgRNA-dependent off-target effects of gene-editing tools, but are time- and labor-intensive to construct. The current study provides a proof-of-concept demonstration of the EGOLD application for a markedly simpler, but thorough, in-depth examination of off-target editing effects. EGOLD can also be used to increase the number of libraries from tens of thousands to millions, enabling the detection of off-target effects under natural conditions, and it can be applied to any cell type, species, or gene-editing tool. The off-target patterns we observed for various gene-editing tools closely matched their on-target base-editing activity (e.g., C/G-to-T/A or A/T-to-G/C conversions; Fig. 2d and Supplementary Fig. S2c). Using this method, we generated comprehensive off-target profiles for several Cas9 variants and base editors, which revealed several important factors influencing their off-target activity.

The data provided by EGOLD also enabled the incorporation of genomic features, especially epigenetic features, in the construction of high-accuracy machine learning-based predictive models. Evidence obtained in the current study indicates that both epigenetic features and chromatin accessibility regulate off-target editing activity. These findings align with those of previous studies that reported that the on-target efficiency of CRISPR/Cas9 is influenced by epigenetic features and other chromatin accessibility features in different genome editing applications, such as base editing^14^ and prime editing^35^. Interestingly, edits involving perfectly matched or single mismatches with the sgRNA appear to be less affected by epigenetic features. However, as the number of mismatched base pairs increases, the correlation between off-target edits and epigenetic features significantly increases. This finding indicates that the determination of off-target edits is more likely to be influenced by epigenetic features and/or chromatin accessibility when the stability of sgRNA–DNA binding is reduced.

In addition to facilitating mechanistic evaluation of contributing factors in off-target data, the epigenetic features we examined here also helped to increase the accuracy of machine learning-based off-target prediction models. In previous studies, machine learning models were typically trained with data derived from lentiviral libraries^33,34^, in which off-target sequences were randomly inserted into the genome and therefore could not provide information about endogenous genome location, epigenetic modifications, or chromatin accessibility. In contrast, the EGOLD data included all of these genomic features, which we found to significantly influence the off-target activity of Cas9 variants and base editors; notably, the incorporation of this data improved the training of predictive models. These results indicate that the chromatin context, such as sequence, epigenetic features, and chromatin accessibility features, of candidate off-target sites should not be overlooked in the development of clinical therapeutic gene-editing applications or off-target prediction model training for gene-editing tools. EGOLD primarily leverages repetitive or sequence-redundant genomic regions. In clinical applications, genome editing is most often directed toward protein-coding genes for the treatment of genetic diseases, and these targets are typically single-copy loci. Although EGOLD can be applied to predict off-target effects on single-copy genes, differences in chromatin context between repetitive regions and protein-coding loci may limit its prediction accuracy. Future incorporation of off-target datasets from protein-coding regions may further refine EGOLD and increase its applicability to clinically relevant targets.

Comparison of editing activity among several Cas9 variants, alone and in combination with TadA-8e, rAPOBEC1, and YE1, using the same target site confirmed that SuperFiCas9 indeed provided the highest fidelity of all the Cas9 variants. We also examined off-targets associated with TadA-8e-nCas9, which has been widely adopted for research and clinical therapy applications, such as PCSK9-related gene therapy, because of its high nucleotide substitution efficiency in in vivo gene editing^36^. However, our results show that TadA-8e-nCas9 can introduce relatively high levels of sgRNA-dependent off-target effects, suggesting that further optimization through closer scrutiny of its off-target pool is still necessary. In addition, we found that fusion with nSuperFiCas9 significantly decreased the number of sgRNA-dependent off-target edits for TadA-8e. Despite the above discovery of its off-target activity, our results suggest that among the tested editors, SuperFiCas9 provides the highest efficiency, with the fewest off-target effects.

Our present study thus shows that compared with other current methods, EGOLD facilitates more comprehensive analysis of sgRNA-dependent off-target effects. In developing the EGOLD library of candidate target sites and associated off-targets, we identified numerous sites in the human genome that might be well suited for assessing both CRISPR/Cas9-based gene-editing tools and traditional editing tools such as ZFN or TALEN. In the future, off-target data from EGOLD can be used with more advanced tools, such as machine learning-based predictive modeling of off-target/on-target activity by different gene-editing tools. In conclusion, these advances present new opportunities for enhancing the precision and safety of gene-editing tools in both research and gene therapies.

## Materials and methods

### Candidate protospacer acquisition

To construct an endogenous sgRNA-dependent off-target detection system, we first designed a pipeline to scan the genome and acquire candidate protospacers. The main criteria for protospacers are multiple potential off-target sites and diverse sequence features. To identify candidate protospacers, we initially scanned the entire genome in both forward and reverse directions for potential target loci in eight main steps: (1) identification of NGG PAM sequences; (2) detection of 20 bp protospacers with a GC content of 40% < 80%; (3) removal of protospacers with completely soft-masked sequences; (4) filtering out protospacers with > 14 candidate sites with 100% identity on the same chromosome; (5) identification and exclusion of protospacers associated with lethal or cell cycle-related genes; (6) removal of simple sequence repeats, such as four identical tandem bases or three tandem repeats of two or three identical bases; (7) identification of editing windows that contain A and C bases; and (8) identification of candidate sites with > 5000 potential off-target sites.

The resulting pipeline has been uploaded to GitHub (https://github.com/offtargetor/EGOLD), and the script supports parameter input. Inputting parameters such as the reference genome, PAM sequence, sgRNA length, PAM position, positions of genes related to lethality and the cell cycle, bin path, starting positions of A and C, window width, and number of CPU threads allows users to automatically generate candidate sgRNAs. Lethal genes and cell cycle-related genes were identified using the GeneCards website (https://www.genecards.org/). To test the pipeline and ensure its usability, we adopted Hg38 as a reference genome to test endogenous candidate sgRNAs for PAMs such as TTTR, NGG, and NGGTTR (https://github.com/offtargetor/EGOLD/results). EGOLD was also used to find endogenous candidate sequences with NGG PAMs in the following genomes: *Sus scrofa* (NCBI:GCA_000003025.6_Sscrofa11.1), *Gallus gallus* (NCBI:GCA_016700215.2_bGalGal1.pat.whiteleghornlayer. GRCg7w_WZ), *Ovis aries* (NCBI:GCA_016772045.2_ARS-UI_Ramb_v3.0), *Bos taurus* (NCBI:GCA_002263795.4_ARS-UCD2.0/GCA_002263795.4_ARS-UCD2.0), *Oryza sativa* ssp. japonica cv. Nipponbare (RiceSuperPIRdb:T2T-NIP), and *Zea mays* (NCBI:GCA_902167145.1_Zm-B73-REFERENCE-NAM-5.0). Owing to the constraints of the seed length in the Blastn alignment algorithm, it was unable to identify candidate sites with a greater number of mismatches. To efficiently identify all potential off-target sites, we developed a C-language script called find-mismatch. The final selected off-target sequences were required to have no more than nine mismatched bases relative to the target protospacers and PAM sequences.

### Plasmid construction

The plasmids for RYCas9, SuperFiCas9, HiFiCas9, HypaCas9, eCas9, OptiCas9, and Sniper2ICas9 were constructed through site-directed mutagenesis. Corresponding base editors, such as TadA-8e, rAPOBEC1, and YE1, were subsequently constructed on the basis of the respective Cas9 variants. Plasmids expressing gRNA were constructed using the pCMV-EGFP-polyA-U6-BbsI-scaffold plasmid as the backbone.

### Cell culture, transfection, and FACS

HEK293T cells were maintained in DMEM supplemented with 10% FBS in a 37 °C humidified incubator with 5% CO_2_. Editing tools and sgRNA expression plasmids were co-transfected using PEI according to the manufacturer’s protocols. At 48 h post-transfection, the cells were washed with PBS and trypsinized using 0.05% trypsin–EDTA. The cell suspension was filtered through a 40-μm cell strainer, and EGFP/mCherry-positive cells were isolated by FACS.

### Evaluation of targeting by candidate sgRNAs

To validate whether candidate sgRNAs could be used to detect sgRNA-dependent off-targets, we randomly selected 10 sgRNAs for target validation. The target site sequences were obtained using 2×Hieff^®^ Ultra-Rapid HotStart PCR Master Mix (with Dye) (Yeasen) through nested PCR. mCherry and GFP double-positive cells were isolated by FACS at 48 h after transfection. Genomic DNA was extracted using a Crude DNA Extraction Kit (for Blood) (Vazyme) according to the manufacturer’s instructions. Target sites were amplified by nested PCR using site-specific primers. PCR was performed for two rounds under the following conditions in each round: 95 °C for 3 min; 30 cycles at 95 °C for 15 s, 59 °C for 15 s, and 72 °C for 15 s; and a final extension at 72 °C for 5 min. PCR products were subsequently purified using a universal DNA purification kit (TIANGEN) following the instructions accompanying the kit. The amplicons were ligated to adapters, and sequencing was performed on the DNBSEQ-T7 platform.

### Transfection of candidate sgRNAs for WGS

Cells were harvested 48 h post-transfection, and EGFP^+^/mCherry^+^ double-positive cells were sorted by FACS. Genomic DNA was isolated using a TIANamp Genomic DNA Kit (TIANGEN) according to the manufacturer’s instructions, and WGS was subsequently conducted on the DNBSEQ-T7 platform at close to 40× coverage, resulting in approximately 120 Gb of data for each individual sequencing event. Specifically, for site 1, three independent biological replicates of cells transfected with reporter plasmids (mCherry only) without any gene editing were subjected to WGS as negative controls.

### Off-target detection

Sequencing data were processed using SOAPnuke^37^ to obtain clean data with default settings. The clean read data were subsequently aligned to the reference genome using BWA-MEM^38^. Variant calling was conducted with Mutect2 in the GATK4 package (v4.2.0.0) using the following commands: gatk --java-options “-Xmx20g” Mutect2 -R Hg38.fa -I HEK293T.bam -I editor.bam -tumor editor -normal HEK293T --native-pair-hmm-threads 5 -O editor.vcf. Subsequent filtering was performed as follows: GATK FilterMutectCalls: gatk FilterMutectCalls -R Hg38.fa --max-events-in-region 5 --min-allele-fraction 0.01 -V editor.vcf -O editor.filter.vcf. InDels such as AA/TT- > GG/CC, AAA/TTT- > GGG/CCC, CC/GG- > TT/AA, or CCC/GGG- > TTT/AAA were defined as single-nucleotide mutations. The filtered results were processed with custom scripts to extract mutations at potential off-target sites. On-target sites had a PAM sequence of NGG and matched the sgRNA, while all others were potential off-target sites. Custom scripts were used to analyze sequence variations, mismatches, and PAM sequences at off-target sites.

### Obtaining epigenetic features and chromatin accessibility features

Epigenetic features and chromatin accessibility status were obtained from bedgraph files downloaded from the ENCODE database (Supplementary Table S4). Transcriptomic data were aligned to the Hg38 reference genome using STAR, followed by read count calculation using HTSeq-count^39^. Subsequently, the FPKM values were calculated using GenomicFeatures software^40^. The target region was set as the 23 bp target sequence plus 15 bp upstream and 12 bp downstream (i.e., 50 bp total). We used a custom script to extract values that intersected with the 50 bp region and obtain an average value. DNA methylation values were determined by the number of methylation sites within this 50 bp region. FPKM values were extracted from genes that intersected with the 50 bp region. The extracted feature values were then defined as the score.

### Development and validation of off-target prediction models

On the basis of the off-target information of 17 gene-editing tools in HEK293T cells obtained in the previous section, we constructed a machine learning model for off-target prediction at sgRNA-dependent sites. First, we merged the off-target information from three replicate samples. We grouped the off-target editing characteristics into 75 training input features, including 62 features related to mutation information, such as mismatch details and mutation patterns in off-target editing sequences, and 13 features related to epigenetic features and chromatin accessibility features from the ENCODE database. These features were initialized with the same weight settings and subsequently utilized in classification and regression tasks using the random forest method to identify optimal features within a randomized subset of inputs and to construct improved prediction models. The dataset was divided into a training set (80%) and a testing set (20%). Various Cas9 variants and base editors were subjected to iterative testing with different combinations of hyperparameters (n_estimators: [50, 100, 150, 200, 250, 500], max_depth: [3, 5, 7, 9, 12, 15], learning_rate: [0.001, 0.01, 0.05, 0.1, 0.2], subsample: [0.6, 0.8, 1.0], and colsample_bytree: [0.6, 0.8, 1.0]), and the best-performing model was then saved. The model was developed using Python v3.8.18, with pandas utilized for data reading and saving. To split the training data, sklearn.model_selection from the scikit-learn library was employed. The training process took place on a cluster running Ubuntu 20.04.6 LTS (GNU/Linux 5.15.0–100-generic x86_64) equipped with an Intel(R) Xeon(R) Gold 6336Y CPU @ 2.40 GHz. The best-trained models were uploaded to https://github.com/offtargetor/EGOLD/models. The weights of the model input features were subsequently extracted to investigate patterns of off-target effects.

### Statistical analysis

Statistical analyses in this study were conducted using R v4.1.0 (https://www.r-project.org). The Wilcoxon test was used for all tests between groups, with a significance level of *P* < 0.05.

## Supplementary information

Supplementary Figures

Supplementary Tables S1-10

## Data Availability

The raw data were deposited in the National Center for Biotechnology Information (NCBI) Sequence Read Archive database with accession number PRJNA1103429.
