# Supplementary material for: Decoding the role of chromatin context in the off-target effects of CRISPR gene editing with EGOLD
Source: Cell Discov. 2026 May 13;12:32. doi: 10.1038/s41421-026-00889-2 (PMC13172344; doi:10.1038/s41421-026-00889-2)
Supplement: Supplementary file 1 — Supplementary Figures [file 41421_2026_889_MOESM1_ESM.pdf]

Supplementary Figures and Figure Legends

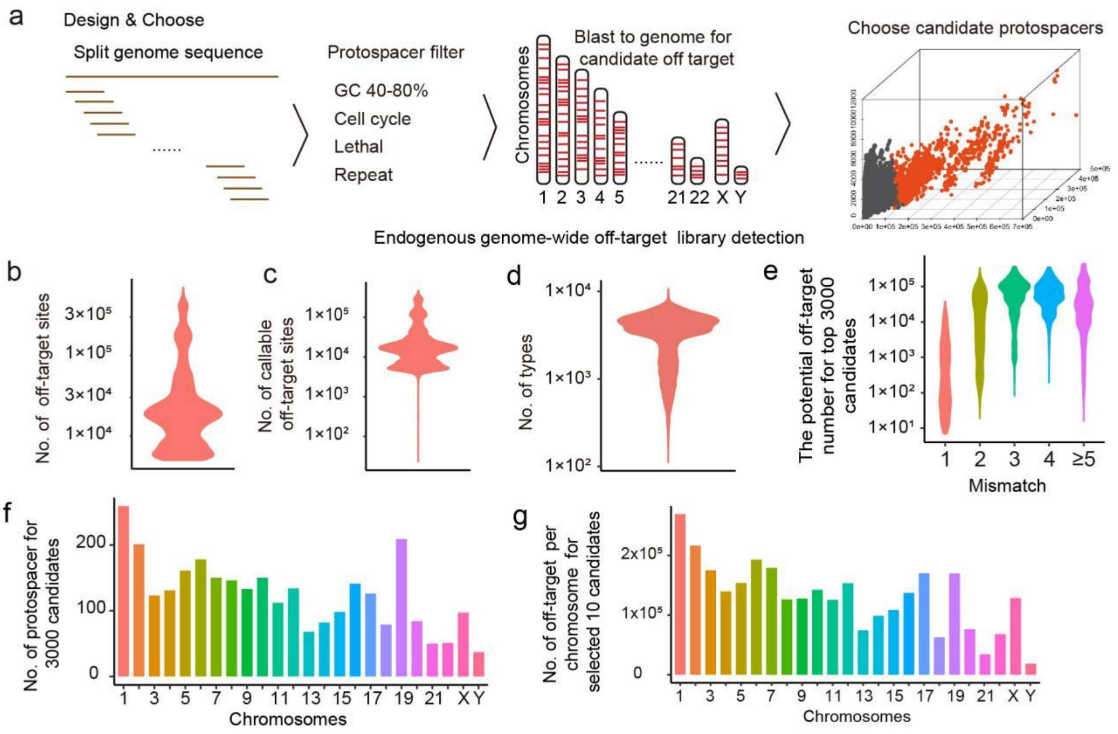

Supplementary Fig. S1 Design of endogenous genomics-wide off-target library detection method. a Schematic diagram of the Endogenous Genome-Wide Off-Target Library Detection sequencing (EGOLD), including genome-wide scan for candidate protospacers. **b** The distribution of off-target numbers for the candidate protospacers. **c** The distribution of callable off-targets (i.e., they were callable in the analysis process) abundance for the candidate protospacers. d, The number of target types for all candidate protospacers. **d** The percentage of callable for all candidate protospacers. **e** The distribution of counts for different possible mismatches for the top 3,000 candidate protospacers. 1, 2, 3, 4, and ≥5 indicate one, two, three, four, or ≥5 mismatches, respectively. **f** The distribution of sites for the top 3,000 candidate protospacers. **g** The distribution of off-target counts for the 10 selected candidate protospacers.

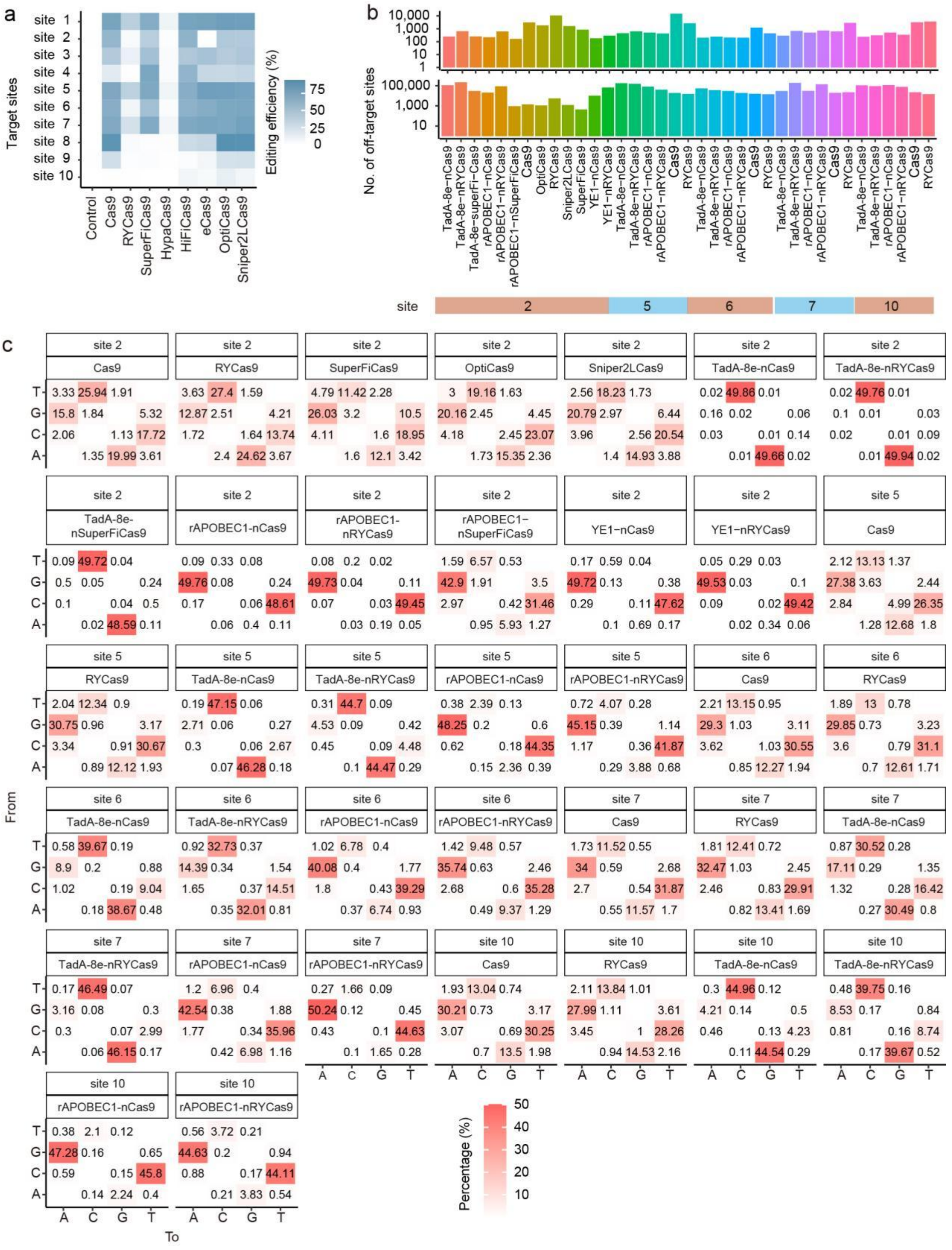

Supplementary Fig. S2 The results of EGOLD. a Determination of targeting efficiency for the selected 10 sgRNAs by deep sequencing. **b** Number of sgRNA-dependent InDels or SNVs off-targets for 17 editing tools targeting the site 2, 5, 6, 7, and 10. **c** Heatmap illustrating the pattern of single-nucleotide mutations occurring in the 13 editing tools of site 2, 5, 6, 7, and 10. "From" indicates the base on the reference genome, while "To" indicates the base after mutation.

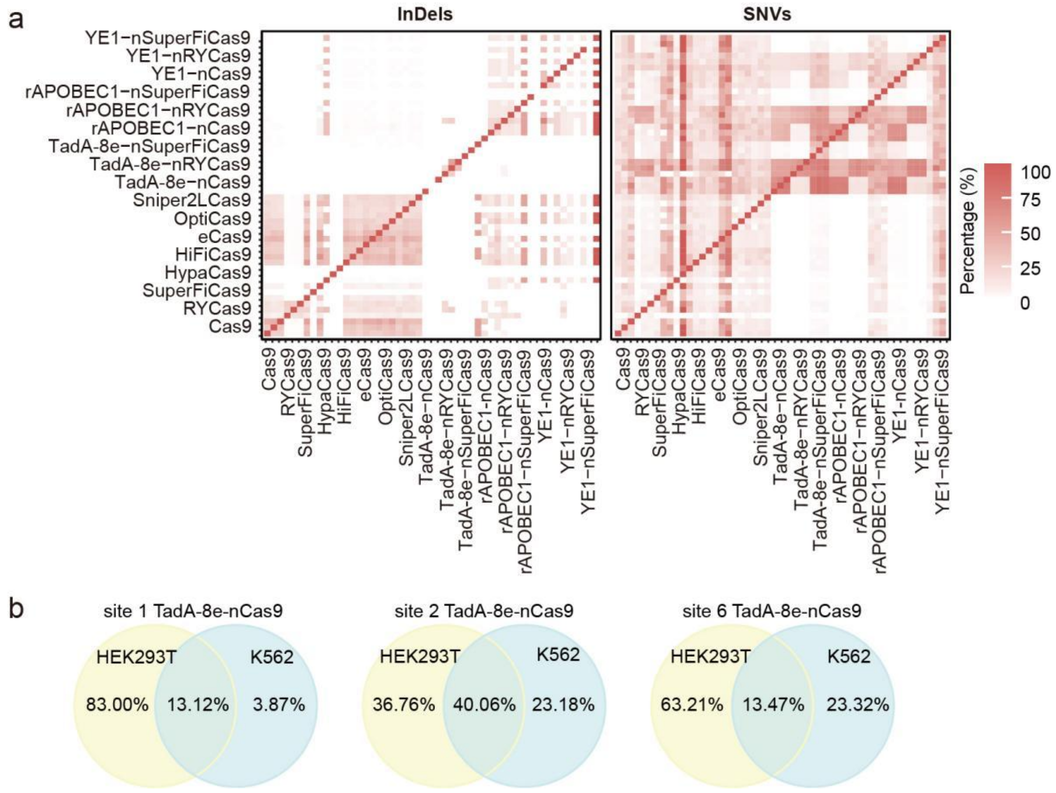

Supplementary Fig. S3 Overlap of off-targets across editors and cell lines. a Heatmap of overlapping off-targets among the 17 editing tools of site 1. Three replicates per gene editing tool. "No. of" means "The number of". **b** Venn diagram of off-target overlap between HEK293T and K562 cell lines.

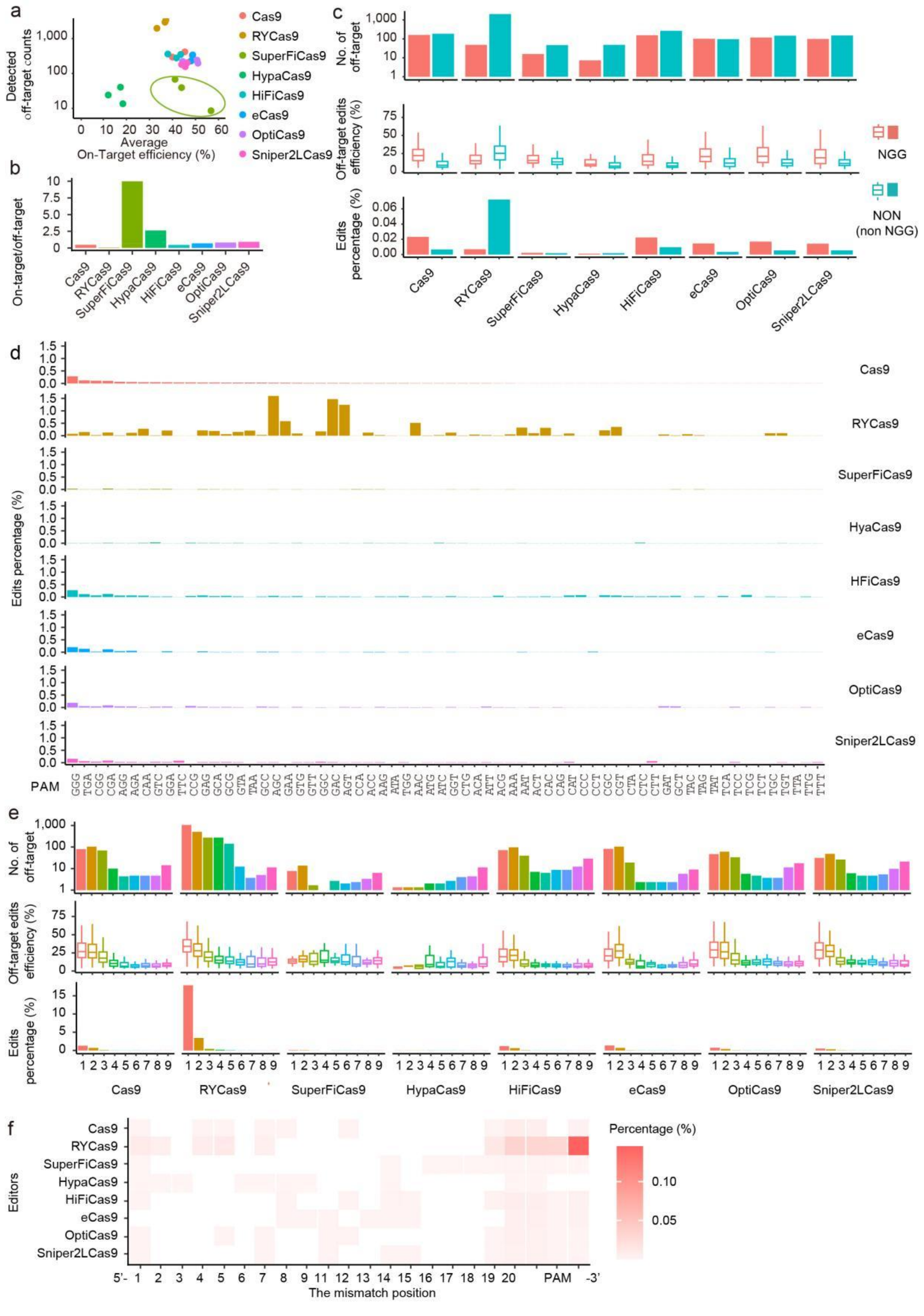

Supplementary Fig. S4 Parallel comparison of off-target effects of various Cas9 variants using EGOLD in site 1. a Distribution of on-target efficiency and off-target counts for the editing tools. b The normaliztion of on-target efficiencies by off-target frequencies. **c** "NGG" and "non-NGG" at off-target sites, including counts, editing efficiency, and off-target ratio for different PAMs. Off-target ratios were calculated using the following formulas: (Number of "NGG" off-targets / Total number of "NGGs" in all potential off-target sites); (Number of "non-NGG" off-targets / Total number of "non-NGGs" at all potential off-target sites). **d** Editing percentage of off-

targets at different PAM for Cas9 variants. Editing percentage were calculated using the following formulas: (Number of PAM type off-targets / Total number of PAM type in all potential off-target sites). **e** Number, efficiency, and ratio of off-targets with different mismatches, calculated as follows: (Number of off-targets with the mismatch / Total frequency of this mismatch in all potential off-target sites). **f** The top ten sites with the largest percentage of off-targets with mismatches. Percentage of off-targets with mismatches at different positions of the guide RNA and PAM, calculated as follows: (Number of off-targets with the mismatch at the position / Total number of mismatches occurring at that position). "No. of" means "The number of".

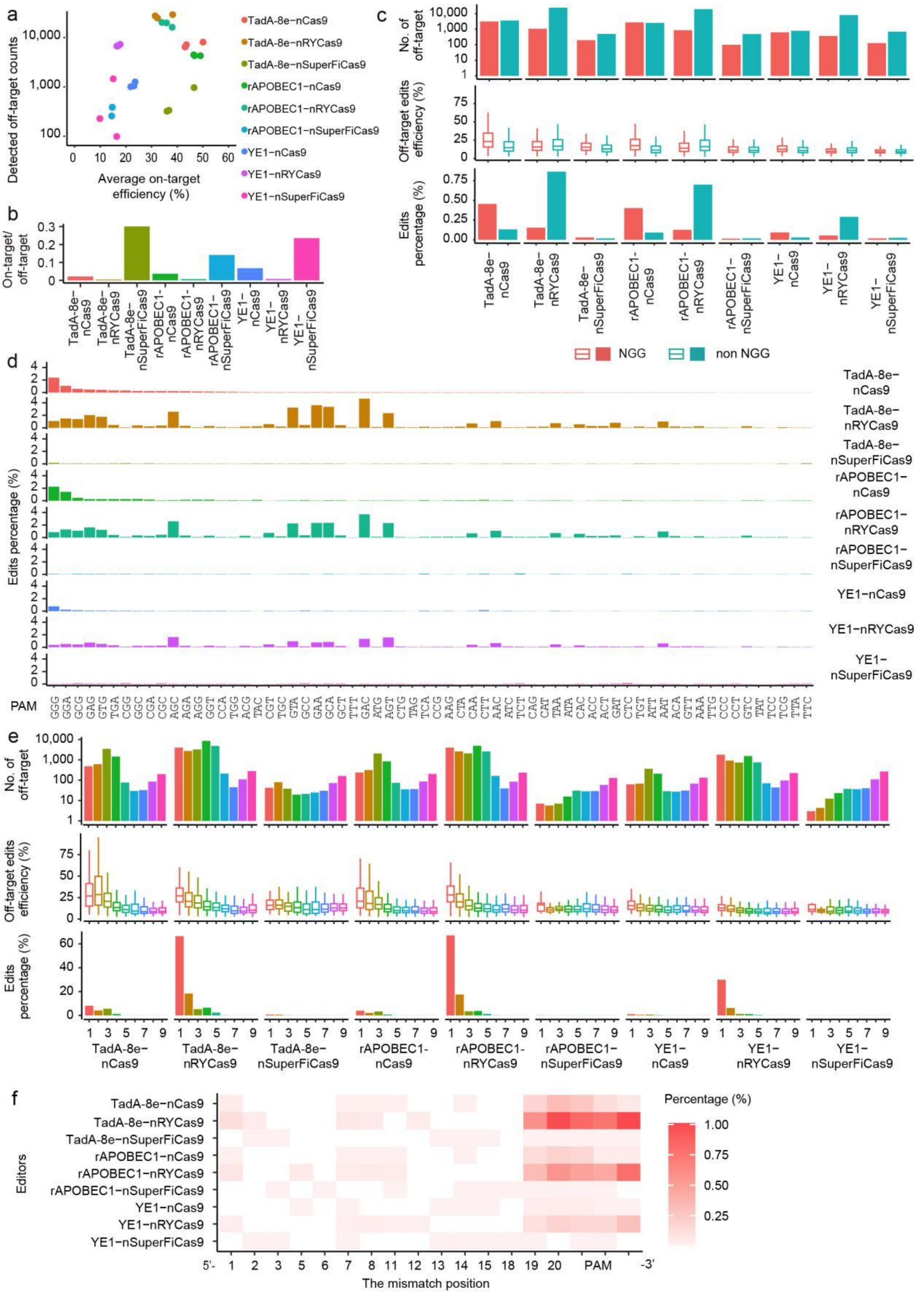

Supplementary Fig. S5 Parallel comparison of off-target effects of different base editors using EGOLD in site 1. a On-target efficiency of three deaminases in combination with three Cas9 nickase variants. b The normaliztion of on-target efficiencies by off-target frequencies. **c** "NGG" and "non-NGG" at off-target sites, including counts, editing efficiency, and off-target ratio for different PAMs. Off-target ratios were calculated using the following formulas: (Number of "NGG" off-targets / Total number of "NGGs" in all potential off-target sites); (Number of "non-

NGG” off-targets / Total number of “non-NGGs” at all potential off-target sites). **d** Editing percentage of off-targets at different PAM for base editors. Editing percentage were calculated using the following formulas: (Number of PAM type off-targets / Total number of PAM type in all potential off-target sites). **e** Number, efficiency, and ratio of off-targets with different mismatches, calculated as follows: (Number of off-targets with the mismatch / Total frequency of this mismatch in all potential off-target sites). **f** The top ten sites with the largest percentage of off-targets with mismatches. Percentage of off-targets with mismatches at different positions of the guide RNA and PAM, calculated as follows: (Number of off-targets with the mismatch at the position / Total number of mismatches occurring at that position). "No. of" means "The number of".

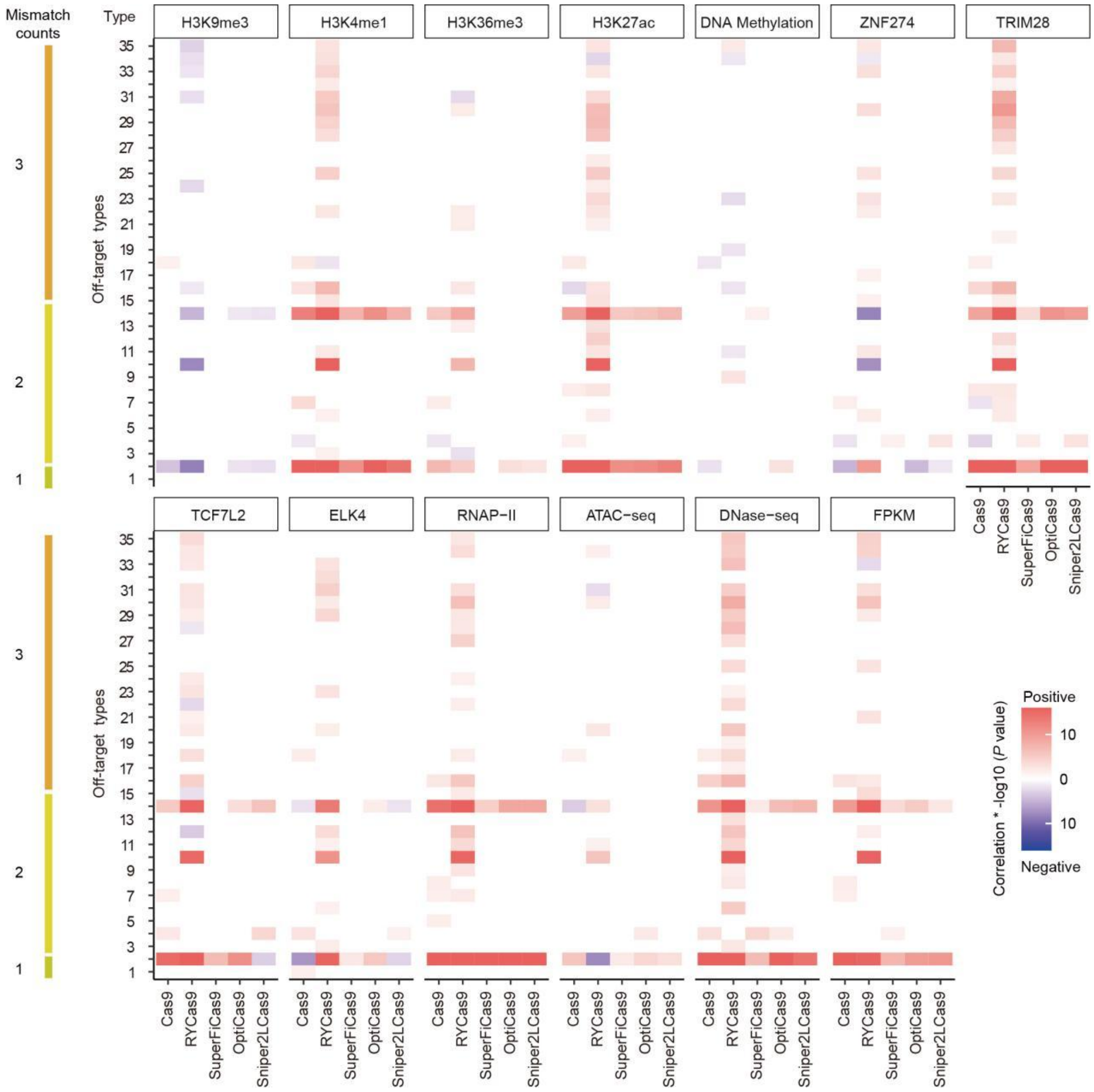

Supplementary Fig. S6 Effects of epigenetic features and chromatin accessibility on off-target activity of Cas9 variants with site 2. Relationship between off-target effects and different epigenetic features or chromatin accessibility for each Cas9-variant gene editor ($P < 0.05$, Wilcoxon test). Red reflects positive correlations; blue reflects negative associations.

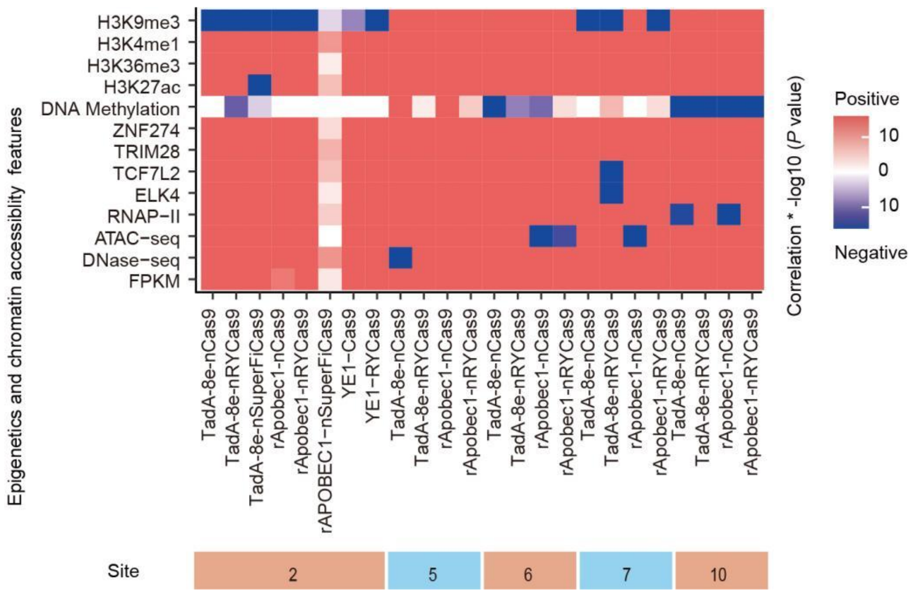

Supplementary Fig. S7 Heatmap of P-values for different epigenetic or chromatin accessibility features in correlation analyses with edited and unedited off-target sites for base editor. Red reflects positive correlations; blue reflects negative associations ($P < 0.05$, Wilcoxon test).

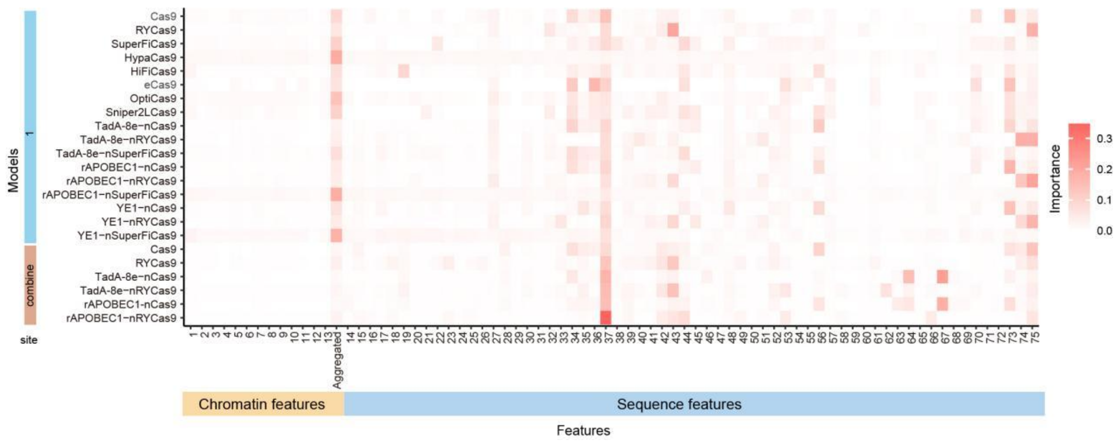

Supplementary Fig. S8 Importance of Features in the Models. X-axis represents feature names, which are listed in Supplementary Table S10. Y-axis represents model names.
